# An Attenuated Cytomegalovirus Vaccine with a Deletion of a Viral Chemokine Gene Is Protective against Congenital CMV Transmission in a Guinea Pig Model

**DOI:** 10.1155/2013/906948

**Published:** 2013-08-20

**Authors:** Michael P. Leviton, Juan C. Lacayo, K. Yeon Choi, Nelmary Hernandez-Alvarado, Andrew Wey, Mark R. Schleiss

**Affiliations:** ^1^Department of Pediatrics, Division of Pediatric Infectious Diseases and Immunology, University of Minnesota Medical School, Center for Infectious Diseases and Microbiology Translational Research, 2001 6th Street SE, Minneapolis, MN 55455, USA; ^2^Food and Drug Administration, Regulatory Review Office, Rockville, MD 20852, USA; ^3^Texas A & M Health Sciences Center, Microbial and Molecular Pathogenesis, College Station, TX 77843, USA; ^4^Biostatistical Design and Analysis Center, Clinical and Translational Science Institute, University of Minnesota, 717 Delaware Street SE, Minneapolis, MN 55455, USA

## Abstract

Development of a vaccine against congenital cytomegalovirus (CMV) infection is a public health priority, but CMVs encode immune evasion genes that complicate live virus vaccine design. To resolve this problem, this study employed guanosyl phosphoribosyl transferase (*gpt*) mutagenesis to generate a recombinant guinea pig CMV (GPCMV) with a knockout of a viral chemokine gene, GPCMV MIP (*gp1*). MIP deletion virus replicated with wild-type kinetics in cell culture but was attenuated in nonpregnant guinea pigs, demonstrating reduced viremia and reduced inflammation and histopathology (compared to a control virus with an intact GPCMV MIP gene) following footpad inoculation. In spite of attenuation, the vaccine was immunogenic, eliciting antibody responses comparable to those observed in natural infection. To assess its protective potential as a vaccine, either recombinant virus or placebo was used to immunize seronegative female guinea pigs. Dams were challenged in the early 3rd trimester with salivary gland-adapted GPCMV. Immunization protected against DNAemia (1/15 in vaccine group versus 12/13 in the control group, *P* < 0.01). Mean birth weights were significantly higher in pups born to vaccinated dams compared to controls (98.7 g versus 71.2 g, *P* < 0.01). Vaccination reduced pup mortality, from 35/50 (70%) in controls to 8/52 (15%) in the immunization group. Congenital GPCMV infection was also reduced, from 35/50 (70%) in controls to 9/52 (17%) in the vaccine group (*P* < 0.0001). We conclude that deletion of an immune modulation gene can attenuate the pathogenicity of GPCMV while resulting in a viral vaccine that retains immunogenicity and demonstrates efficacy against congenital infection and disease.

## 1. Introduction

Human cytomegalovirus (HCMV) is the most common cause of viral congenital infection in the developed world and is estimated to complicate approximately 0.5–2% of pregnancies in the United States and Europe. Congenital infections can cause severe sequelae among neonates including sensorineural hearing loss, cognitive impairments, and mental retardation [1–3]. In the setting of maternal primary infection or reinfection during pregnancy, HCMV can translocate the placental barrier and can cause infection of the developing fetus, with attendant morbidity and occasional mortality [4]. Unfortunately, animal models are of limited usefulness in the study of antiviral and vaccine strategies against HCMV, due to the extreme species specificity of CMVs. It is therefore necessary to study species-specific CMVs in animal models that mimic HCMV congenital infection, in order to evaluate therapeutic and preventive strategies that may ultimately be clinically useful. Among the CMVs of small mammals, the GPCMV has the unique advantage of crossing the placenta, causing infection *in utero* [5, 6]. This feature of the biology of GPCMV makes it ideal for vaccine studies, since congenitally infected pups, like newborn infants, have virus-related morbidity and mortality.

All CMVs encode genes that confer immunomodulatory functions that may impact the efficiency of infection, dissemination, reactivation and persistence in the host (reviewed in [7]). Such immune evasion genes include homologs of chemokines (CKs), G protein-coupled receptors (GPCRs), and modulators of antigen processing and presentation [8–12]. In addition to contributing to modification of host immune responses, some of these viral proteins may play a role in promoting dissemination during acute infection. GPCMV, similar to other CMVs, encodes a number of potential immunomodulatory gene products [13, 14]. The *gp1* ORF encodes one such gene product that has been characterized in previous studies [13, 14]. The protein, GPCMV-MIP, is a member of the CC family of CKs and is most closely related to the macrophage inflammatory protein (MIP) 1*α* family. Previous studies with a recombinant form of this protein demonstrated that specific signaling could be mediated via the hCCR1 receptor and that this interaction could be blocked with human MIP 1*α* in competition experiments [14]. Moreover, migration assays revealed that GPCMV-MIP was able to induce chemotaxis in transfected hCCR1-L1.2 cells. Subsequent studies in a model of virus-induced labyrinthitis, comparing a recombinant GPCMV deleted of the GPCMV MIP gene with wild-type virus, indicated a potential role for this CK in pathogenesis, insofar as the “knock-out” virus demonstrated reduction both in the magnitude of hearing loss and in cochlear inflammation, following direct inoculation of the guinea pig cochlea via the round window [15, 16]. Thus, deletion of this gene appears to substantively attenuate the pathogenicity of the resulting recombinant virus *in vivo*.

Both purified protein subunit vaccines and live, attenuated vaccines have been proposed as strategies to prevent congenital CMV infection [17]. Significant concerns have been raised, however, regarding the deployment of live, attenuated HCMV vaccines in clinical practice. These include the concern that live virus vaccines might establish latency, theoretically putting the vaccine recipient at risk to develop as yet unproven long-term adverse consequences related to HCMV infection, including autoimmune disease, malignancy, and atherosclerosis [18]. In addition, any live virus vaccine must in principle be sufficiently attenuated such that it would not pose any untoward risks when administered to vaccinees, including the setting of inadvertent administration to a pregnant woman. A potential solution to the challenge of developing a safe live-virus vaccine for HCMV is to use recombinant technologies to engineer attenuated vaccines that, by virtue of targeted removal of genes that contribute to immune modulation and/or pathogenesis *in vivo*, could in principle retain immunogenicity while avoiding potential risks. This strategy has been successfully employed in the murine CMV (MCMV) model, where deletions of large segments of the viral genome encoding immune modulation genes result in a virus with essentially complete attenuation and an inability to establish latency, which nonetheless retains immunogenicity and provides protective efficacy as a vaccine [19–21]. However, a limitation of the MCMV model is that it does not allow testing of vaccines for prevention against congenital transmission, because of the inability of MCMV to cross the mouse placenta [22]. Therefore, we sought to test whether a live, attenuated GPCMV vaccine when administrated before conception could engender an immune response sufficient to protect against congenital viral transmission in the guinea pig model. Specifically, these studies were undertaken to test the hypothesis that a GPCMV (*gp1*) MIP deletion virus would, though attenuated, retain the ability to protect against congenital GPCMV infection and disease when used as a preconception vaccine.

## 2. Materials and Methods

### 2.1. Cells and Viruses

**Figure 1:**
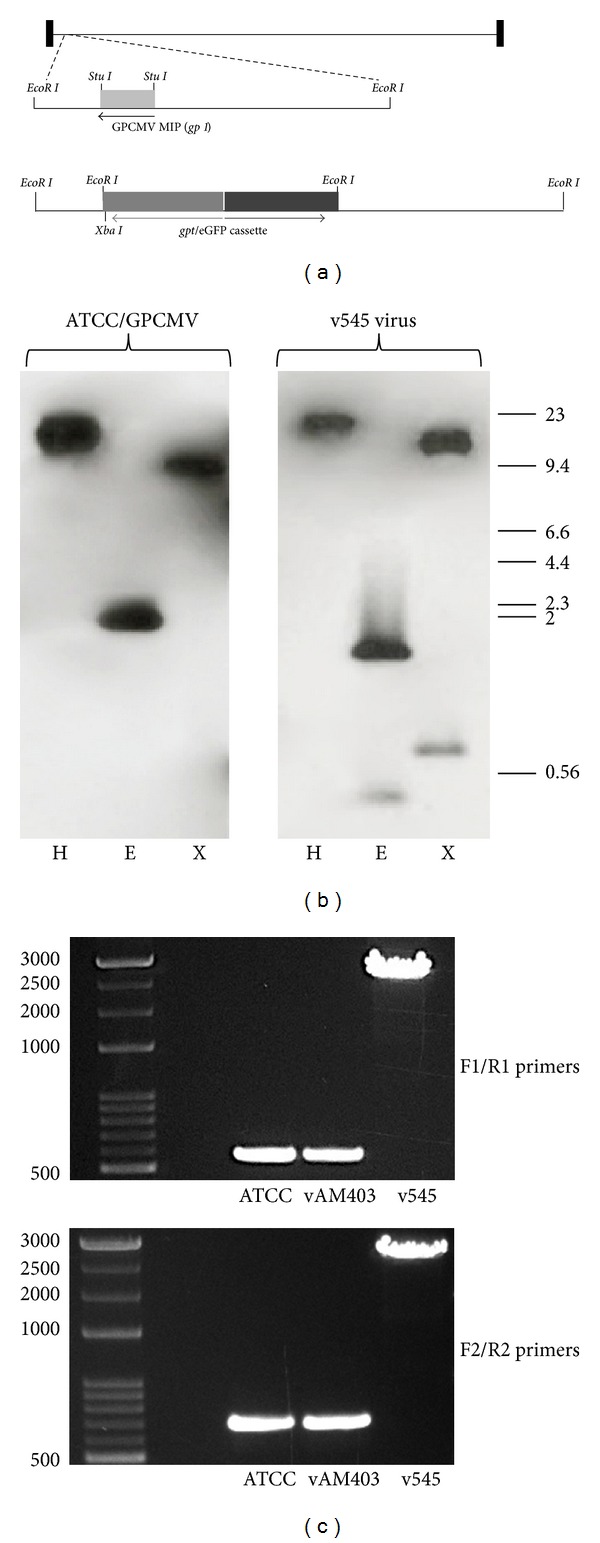
Schematic representation of the GPCMV MIP gene and generation of recombinant virus. (a) Map of GPCMV genome. GPCMV MIP (*gp1* gene) maps to 2.3 kb *EcoR* I fragment near left genome terminus. Knockout of *gp1* was achieved by introduction of *gpt*/eGFP cassette into a *Stu* I collapsed version of plasmid DNA. Following co-transfection of plasmid and viral DNA and *gpt* selection as described in text, a clonal recombinant virus was obtained by limiting dilution. (b) Southern blot analysis of wild-type (ATCC 22122; left panel) and v545 (recombinant) DNA. Probing with pKTS107 probe revealed presence of restriction polymorphisms demonstrating predicted configuration of recombinant virus. H, *Hin*d III digest; E, *EcoR* I digest; X, *Xba* I digest. Molecular weight markers, lamba/*Hin*d III ladder. (c) To further characterize the genome structure of the v545 mutant, viral DNA was analyzed by PCR. The PCR was done using primer pairs cassette F1/R1 (upper panel) and cassette F2/R2 (lower panel) which, respectively, amplify 552 bp and 671 bp products in wild-type GPCMV and vAM403. Insertion in v545 coupled with deletion of MIP gene results in overall shifts in bands to *∼*2.8-2.9 kb, as predicted. Subsequent sequence analysis of gel-purified products confirmed predicted insertion and genome structure of v545 vaccine virus.

GPCMV (strain 22122, ATCC VR682), v545 [15, 16], and vAM403, an enhanced green-fluorescent-protein-(eGFP-) tagged GPCMV [23], were propagated on guinea pig fibroblast lung cells (GPL; ATCC CCL 158) in F-12 medium supplemented with 10% fetal calf serum (FCS; Gibco-BRl), 10,000 IU of penicillin/liter, 10 mg of streptomycin/liter (Gibco-BRL), and 7.5% NaHCO_3_ (Gibco-BRL). For generation of the GPCMV MIP “knock-out” virus, guanosyl phosphoribosyl transferase (*gpt*) mutagenesis was employed, as previously described [23]. A 2.3 kb *EcoR* I fragment containing the GPCMV-MIP was subcloned into pBluescript (+) from a plasmid containing the *Hin*d III “D” fragment, yielding plasmid pKTS 107. A 320 bp *Stu* I collapse, deleting the GPCMV MIP gene, yielded pKTS 534. Following deletion of the vector *Xba* I site by digestion with *Xba* I, Klenow polymerase treatment, and religation (yielding pKTS 536), an *Xba* I linker was inserted into the unique *Stu* I site, yielding pKTS 540. Next, a 2.3 kb *Spe *I fragment from pQ106 [23] containing the *gpt* and enhanced green fluorescent protein (eGFP) cassette was inserted into the *Xba* I site of pKTS 540, yielding pKTS 545 (Figure 1(a)).

Generation of the MIP deletion virus was performed as described elsewhere [15, 16]. For generation of this recombinant virus, plasmid pKTS 545 was cotransfected with viral DNA into GPL cells, and selection was carried out using mycophenolic acid and xanthine as previously described [23]. Following limiting dilution, eGFP-positive wells were identified and 6 rounds of plaque purification were performed under selection, to ensure clonality of the recombinant viral stock. A clonal eGFP-positive recombinant virus was purified and designated as v545; this virus was used for subsequent pathogenesis and vaccine studies.

### 2.2. Characterization of Recombinant Virus

For characterization of recombinant virus, Southern blot analyses were performed. Viral DNA purified from v545 and wild-type (ATCC) GPCMV was subjected to restriction enzyme digestion with *Hin*d III and *Eco*R I, followed by agarose gel electrophoresis. Following transfer to Nytran membranes, DNA probes corresponding to the GPCMV-MIP gene [13] or to the *gpt*/eGFP cassette were labeled with [^32^P] dCTP, using a High Prime kit (Boehringer Mannheim) according to manufacturer's specifications (Figure 1(b)). To further characterize the genome structure of the v545 mutant, viral DNA was analyzed by PCR. The PCR was done using primer pair cassette F1/R1 and cassette F2/R2 which, respectively, amplified 552 bp and 671 bp products in wild-type GPCMV. Primer sequences were cassette F1, 5′-GACCCTCTAACATATCGGAG-3′; cassette R1 5′-AAGAACATGGCTGTCCGCTA-3′; cassette F2 5′-TTCTCTCACGTTGAGCGCAT-3′ cassette R2 5′-CCTATCGATACGTGGATACG-3′. The PCR reaction was performed in a total volume of 50 *μ*l using GoTaq long PCR Master Mix (Promega laboratories) and 1.0 *μ*M primers. The conditions for the PCR were initial denaturation at 95°C for 2 min, followed by 95°C for 30 s, 56°C for 30 s, 72°C for 3 min for a total of 35 cycles, and elongation at 72°C for 10 min. The PCR product (9 *μ*L) was subjected to electrophoresis in a 0.7% agarose gel (Figure 1(c)).

Rescue virus was generated as previously described [24]. Briefly, 1 *μ*g of v545 DNA was cotransfected with 20 *μ*g of rescue plasmid (pKTS 107) onto GPL cells. Rescued, eGFP-negative viral plaques were picked and subject to further rounds of plaque purification by limiting dilution. Rescue of GPCMV-MIP was confirmed by PCR and by restriction profile analysis (data not shown). 

**Figure 2:**
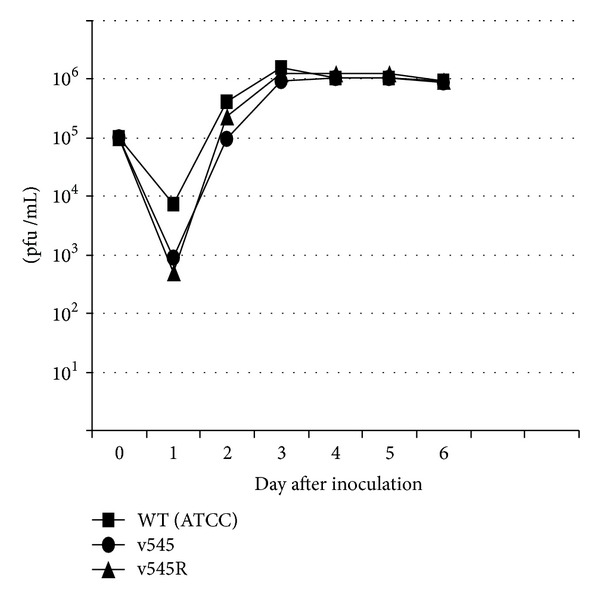
One-step growth curve analysis of recombinant virus v545. Wild-type (ATCC 22122), recombinant (v545) and rescuant (v545R) viruses were used to infect confluent cell culture monolayers at an m.o.i. of 0.5 pfu/cell. After absorption for 1 h at 37°C, the cells were washed with media and a zero time point was harvested. The remainder of the sample was incubated at 37°C with additional time points taken at indicated times. The viral titer of each time point was determined by plaque titration assay on GPL cells.

One-step growth curve analyses were conducted to compare wild-type (GPCMV ATCC, 22122), recombinant, and rescued viruses. Clonal stocks were used to infect confluent monolayers at an m.o.i. of 0.5 pfu/cell. After adsorption for 1 h at 37°C, the cells were washed with media and a zero time point was harvested. Additional time points were obtained at 24, 48, 72, 96, 120, and 144 h after inoculation, and the viral titer of cell culture supernatant obtained at each time point was determined by plaque titration assay on GPL cells (Figure 2).

### 2.3. Animal Studies of Recombinant Viruses following Systemic and Footpad Inoculation

Hartley guinea pigs were purchased from Elm Hill Laboratories (Chelmsford, MA, USA). Strain 2 guinea pigs were maintained in the vivarium of the University of Minnesota Medical School. Animals were housed under conditions approved by the American Association of Accreditation of Laboratory Animal Care, in accordance with and following approval from the Institutional Animal Care and Use Committee at the University of Minnesota. 

**Figure 3:**
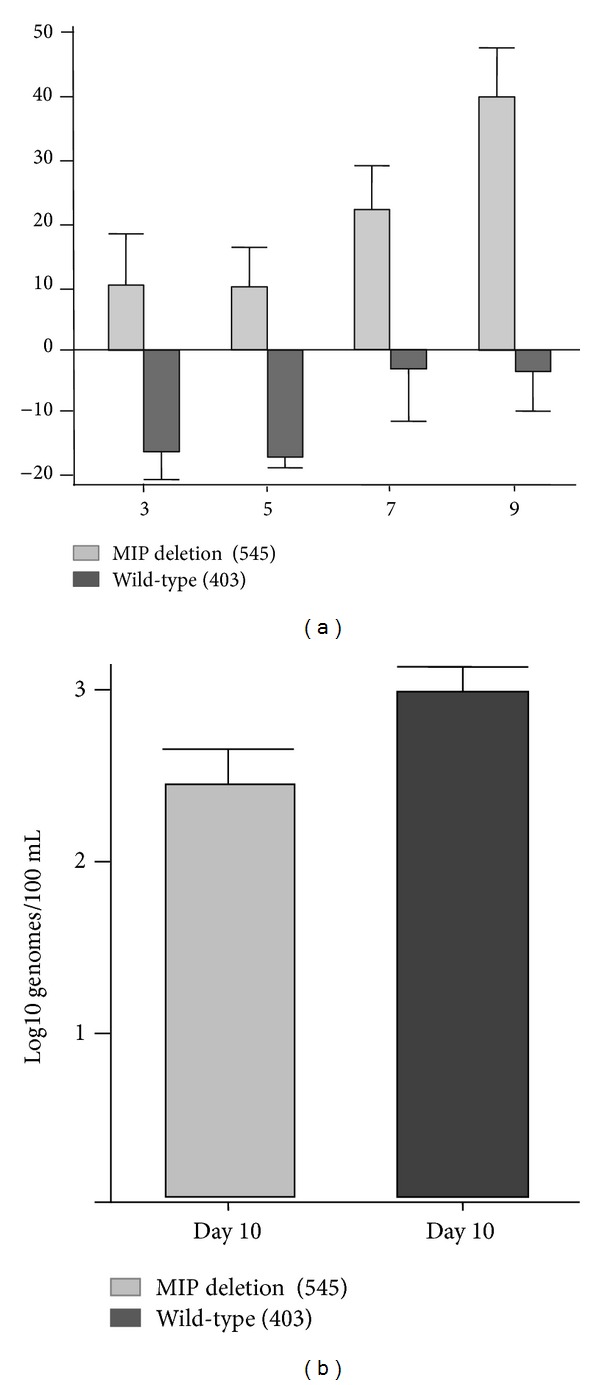
The v545 virus is attenuated in strain 2 guinea pigs. (a) Weight loss and DNAemia profiles were compared following inoculation of young, nonpregnant guinea pigs with v545 and control vAM403 virus. Animals inoculated with v545 gained weight throughout this postinoculation time period (mean weight gain at day 9, 40 grams) compared to animals infected with vAM403 (weight loss of 5 grams; *P* < 0.01). (b) Animals challenged with v545 virus demonstrated reduced DNAemia measured at day 10 (mean viral load, 2.5 log_10_ genomes/mL versus 3.0 log_10_ genomes/mL in vAM403 group; *P* < 0.05).

**Figure 4:**
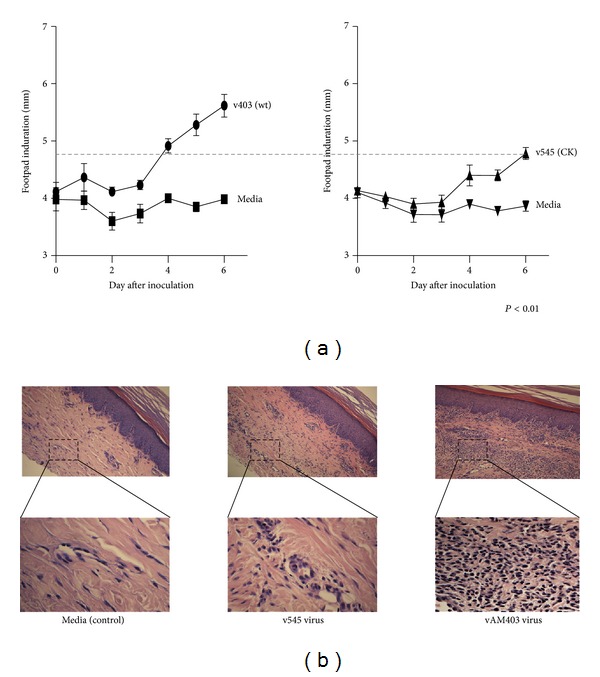
The v545 virus is attenuated in a footpad inoculation model. Using calipers, differences between wild-type (vAM403) and CK deletion virus (v545) were noted following footpad inoculation, beginning at day 3 and progressing through day 6. The level of swelling produced by the vAM403 virus was noted to be ~20% above preinoculation levels, compared to the results obtained with v545 (*P* < 0.01). All swelling resulted from the effects of virus in the inoculum because inoculation of an identical volume of tissue culture medium alone failed to elicit any response (controls, Figure 4(a)). Feet were collected from sacrificed guinea pigs at day 6 after inoculation, and midline longitudinal sections were prepared. Examination by light microscopy at both low and high power (×10 and ×60; Figure 4(b)) revealed substantially larger amounts of both cellularity and edema in animals inoculated with vAM403 virus (Figure 4(b), right panel), containing the GPCMV MIP ORF, than in v545 virus-inoculated animals (Figure 4(b), middle panel) or in media-inoculated control (Figure 4(b), left panel).

The v545 recombinant had been previously demonstrated to have an altered pathogenesis in guinea pigs compared to a similar recombinant, vAM403 (which has the eGFP/*gpt* cassette inserted into a noncoding region of the GPCMV genome), following intracochlear inoculation [15, 16]. To further characterize the impact of deletion of the GPCMV MIP on the *in vivo* pathogenesis of infection, viremia (Figure 3) and footpad inoculation studies (Figure 4) were conducted in nonpregnant, weanling animals. For viremia studies, young strain 2 guinea pigs (*n* = 6/group) were inoculated by intraperitoneal route with 1 × 10^7^ pfu of either vAM403 or v545 virus (Figure 3(a)). The vAM403 virus was used as a control, rather than the v545 rescue virus, since it (like vAM403) expressed eGFP and also had a similar insertion into the viral genome. Animals were then monitored every other day for nine days following infection for weight loss or gain. On day 10, animals were bled for analysis of systemic viral load by PCR (Figure 3(b)). To evaluate inflammation at a local site of primary infection, additional adult animals (*n* = 6) were inoculated in the footpad with 5 × 10^4^ pfu of either vAM403 virus or v545 virus. For each group, media alone was inoculated into the contralateral paw. Foot thickness was measured using a digital caliper as described elsewhere [25], daily for 6 days (Figure 4(a)), in a coded, blinded fashion. On day 7, animals were sacrificed and tissues harvested, formalin fixed, and decalcified; after embedding in paraffin, 5 *μ*m sections were cut and stained with hematoxylin and eosin to evaluate for histopathology and evidence of inflammation (Figure 4(b)).

### 2.4. Vaccine/Challenge Studies

Hartley strain guinea pigs were purchased, as previously noted, from Elm Hill laboratories. All animals (*n* = 15/group) were determined to be GPCMV seronegative prior to vaccination by ELISA. Animals were immunized twice, with an interval of 3 weeks between doses, with 5 × 10^4^ pfu of v545 vaccine, by subcutaneous route in a total volume of 1 mL. Control animals received an identical volume of phosphate-buffered saline. Bleeds were performed once a week for 6 weeks on all guinea pigs following vaccination for both quantitative PCR analysis of viral load and for serum analysis. After 6 weeks, bleeds were performed every other week until the conclusion of the study. 

Three weeks following the second immunization, animals were mated with seronegative male Hartley guinea pigs. Mating persisted until abdominal palpitation confirmed pregnancy. Approximately 4 weeks before delivery, pregnant dams were challenged with a subcutaneous injection of 1 × 10^6^ pfu/mL of a virulent, salivary gland-derived GPCMV workpool. Bleeds were performed in infected dams on days 5, 10, and 15 after challenge for quantitative PCR analysis of viral load and for plasma analysis. Upon delivery, pups were weighed and live-born pups sacrificed within 96 hours of birth and blood obtained for quantitative PCR analysis of viral load. Lung, liver, spleen, and placenta were also extracted for evaluation of organ pathology and quantitative PCR analysis of viral load.

### 2.5. Immunologic and Virologic Studies

**Figure 5:**
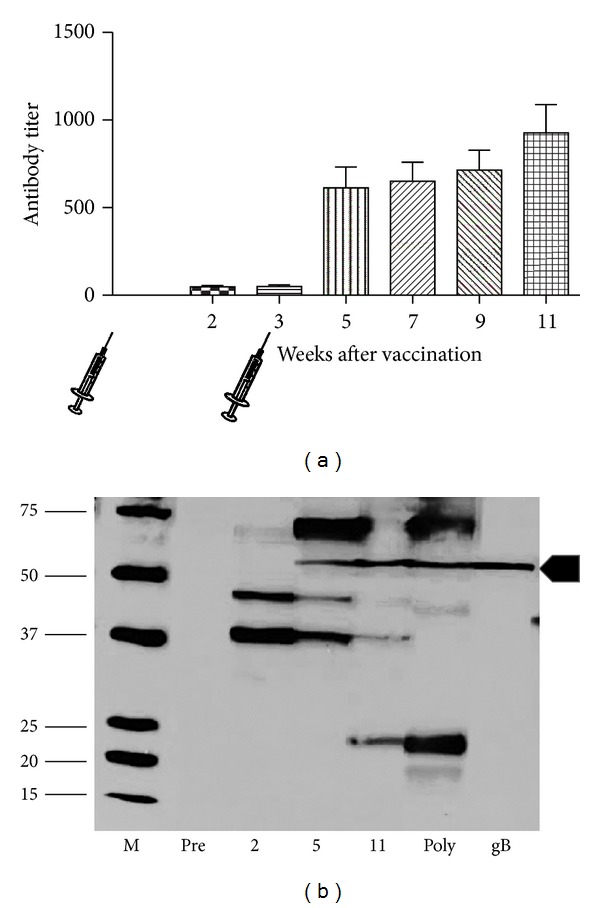
Immunogenicity of v545 attenuated vaccine. (a) ELISA profile following vaccination. Animals were immunized twice, with an interval of 3 weeks between doses, with 5 × 10^4^ pfu of v545 vaccine, by subcutaneous route of inoculation. Control animals received an identical volume of phosphate-buffered saline. Bleeds were performed at regularly intervals, as described in the text, on all guinea pigs following vaccination for serum ELISA analysis. Selected sera were also used in western blot assay as indicated in panel (b). Data shown represents the mean ± SD reciprocal ELISA titer for the vaccine group at each time point. (b) Representative profile of response from vaccinated dam using viral particles in western assay. Marker lane is indicated (M). Lanes 1–4, preimmune sera and sera from weeks 2, 5, and 11 following vaccine. Lane 5, western profile of high-titer anti-GPCMV antisera. Lane 6, western blot with anti-GPCMV gB polyclonal antibody. Immunized animals demonstrate anti-gB antibodies (arrowhead) in addition to antibody to other GPCMV polypeptides. Initial response targets virion proteins of ~35 and 45 kDa noted at 2 weeks after vaccination, followed by responses to gB and a ~70 kDa protein following second immunization at 5-week time point. Last serological responses to appear target proteins of ~22 kDa, noted at 11-week time point.

ELISA analysis (Figure 5(a)) was performed as previously described [26], with titer determined by limiting dilution assay (initial dilution, 1 : 80). A positive result was determined by calculating the reciprocal of the highest dilution that produced an absorbance of at least 0.1 and twice the absorbance observed using a negative control antigen. Plates were read at a wavelength of 450 nm using the SpectraMax M2 Spectrophotometer (Molecular Devices) and the ScanMax Pro program. Western blot analysis was performed on serum collected from a subset of guinea pigs from the vaccine and control groups (Figure 5(b)). Serum was collected before vaccination from each experimental group (preimmune) and at 2, 3, 5, 7, 9 and 11 weeks, as outlined schematically in Figure 5. For western blot studies, virus particles were subjected to SDS-PAGE and were then transferred onto nitrocellulose membranes by electroblotting. Membranes were blocked using membrane blocking agent (GE Healthcare) resuspended in Tris-buffered saline plus 0.5% Tween (TBST) and then incubated for 3.5 hours with the aforementioned collected sera (1 : 800). Along with serum samples obtained from the above time points, a gB-specific polyclonal rabbit antiserum (1 : 500) and a high-titer guinea pig polyclonal antiserum (1 : 10,000) were used as controls [27]. After washing in TBST, blots were incubated with either a horseradish peroxidase-conjugated rabbit anti-guinea pig antiserum (1 : 10,000; Santa Cruz Biotechnology) or a horseradish peroxidase-conjugated donkey anti-rabbit antiserum (1 : 10,000; Santa Cruz Biotechnology) for 2 hours at 27°C. Antibody binding was then detected using the ECL Western Blot Detection Kit (GE Healthcare), followed by autoradiography (Hyblot CL; Denville Scientific, Inc.). 

For detection of viremia following intraperitoneal inoculation of strain 2 guinea pigs (Figure 3), a quantitative, competitive PCR assay was employed, as described elsewhere [28]. For congenital transmission comparisons and for assessment of viral load in pregnant animals, real-time PCR was performed. DNA was extracted from 200 *μ*L citrated whole blood using the MagNA Pure LC System (Roche) or 150 *μ*L of 10% tissue homogenate using the QIAxtractor (QIAGEN). The GPCMV gB-specific primer pair LCF1 (5′-CTTCGTGGTTGAACGGG-3′) and LCR1 (5′-GTAGTCGAAAGGACGTTGC-3′) were utilized for the real-time PCR assay. The PCR reaction was performed in a 20 *μ*L volume reaction as specified by the LightCycler FastStart DNA Master HybProbe reaction mix (Roche Diagnostics). GPCMV gB-specific hybridization probes were used for detection (LCPG 5′-TGGTGACCTTCGTTACCAATCCGTTTGGA-F; LCPR 5′R640-CTTCGTGGTGTTCCTGTTCTGCGT-P). PCR was performed using the LightCycler 480 real-time PCR System (Roche) under the following conditions: initial denaturation at 95°C for 10 min, followed by 95°C for 10 s, 54°C for 15 s, 72°C for 15 s for a total of 50 cycles, followed by melting curve analysis at 95°C for 1 min and 45°C for 1 min and ending at 85°C, and then a final hold step at 40°C. Data were analyzed with the Light-Cycler Data Analysis Software (version 1.0; Roche) using standard curves generated using serial dilutions of plasmid and viral DNA at known concentrations. The magnitude of DNAemia was expressed as the total number of genome copies per mL of blood or total number of genome copies per *μ*g of tissue. 

### 2.6. Statistical Comparisons

Antibody titers were compared using the paired Student's *t*-test. Parametric variables were compared by ANOVA. Nonparametric variables were compared using the Mann-Whitney *U* test or the paired Wilcoxon test. The proportion of live-born pups, infected pups, and the birth weights were compared/calculated using generalized linear mixed models (GLMM) to account for within-litter variability [29]. Statistics were analyzed using the Prism 5.0 software package (GraphPad Software, San Diego) and R v2.13.0.

## 3. Results

### 3.1. MIP Gene Deletion Does Not Modify Viral Replication in Cell Culture but Attenuates GPCMV Infection and Dissemination in Guinea Pigs

The GPCMV MIP gene was deleted from the viral genome using a well-established model of selection for recombinant virus, *gpt* selection. The genome structure of the recombinant virus, v545, was verified by Southern blot study (Figure 1(b)). A GPCMV-MIP-specific probe was found to hybridize with viral DNA purified from GPCMV (ATCC) DNA, but not DNA purified from v545. However, a *gpt*/eGFP-specific probe was found to hybridize with DNA purified from v545, but not ATCC DNA. Moreover, insertion of this cassette into the GPCMV genome generated novel *EcoR* I restriction polymorphisms, which could be demonstrated both with GPCMV-specific probes and *gpt*/eGFP-specific probes. To confirm the Southern blot observations, and also to confirm that potential adjacent ORFs were not modified, PCR was performed (Figure 1(c)), followed by sequence analysis. This confirmed the predicted orientation and insertion into the GPCMV genome. During the course of these studies, it was noted by Inoue and colleagues that two types of strains of GPCMV are present in virus stocks obtained from ATCC: GPCMV/full, and GPCMV/del, containing a 1.6 kb deletion in a locus encoding the GPCMV homologs of the HCMV UL128-131 complex [30]. We noted by PCR that both v545 and vAM403 had a GPCMV/del genome variant, presumably due to prolonged passage of ATCC virus in fibroblast cells (data not shown). We conclude based on these analyses that v545 has a targeted deletion of GPCMV MIP, but otherwise an intact genome structure, compared to the GPCMV/del ATCC variant. 

Replication kinetics of v545 in cell culture were compared to that of wild-type (ATCC) GPCMV and another eGFP-tagged recombinant. In these experiments, one-step growth curve analyses were performed with v545; ATCC virus; a v545 rescuant; and a previously described recombinant GPCMV with the *gpt*/eGFP cassette inserted in a noncoding region of the viral genome, vAM403 [23]. These comparisons revealed that deletion of the GPCMV MIP had no impact on the replication of GPCMV in cell culture (Figure 2). 

Previously, we reported on the reduced pathogenesis of the v545 mutant virus in a direct inoculation model of cochlear pathogenesis and hearing loss [15, 16]. It was therefore of interest to further examine the impact of deletion of the GPCMV MIP ORF on the pathogenesis of infection. Weight loss and DNAemia were compared following inoculation of young, nonpregnant guinea pigs with v545 (MIP deletion virus; Figure 3). Animals inoculated with v545 gained weight throughout this postinoculation time period (mean weight gain at day 9, 40 grams) compared to animals infected with vAM403 (weight loss of 5 grams; *P* < 0.01). In addition, animals challenged with v545 virus demonstrated a significant reduction in the magnitude of DNAemia measured at day 10 (mean viral load, 2.5 log_10_ genomes/mL versus 3.0 log_10_ genomes/mL in vAM403 group; *P* < 0.05). 

To further characterize the biology of wild-type and deletion virus in guinea pigs, a footpad inoculation model was employed, as previously described for analyses of other rodent CMVs [25, 31]. Using this model, differences between wild-type (vAM403) and CK deletion virus (v545) were noted beginning at day 3, and progressing through day 6, when the level of swelling produced by the vAM403 virus was noted to be ~20% above preinoculation levels, compared to the results obtained with v545. All swelling resulted from the effects of virus in the inoculum because inoculation of an identical volume of tissue culture medium alone failed to elicit any response (controls, Figure 4(a)). In order to further investigate cellular infiltrates in response to GPCMV MIP, feet were collected from sacrificed guinea pigs at day 6 after inoculation, and midline longitudinal sections were prepared from formalin-fixed, paraffin-embedded blocks. Examination by light microscopy at both low and high power (×10 and ×60; Figure 4(b)) revealed substantially larger amounts of both cellularity and edema in animals inoculated with vAM403 virus, containing the GPCMV MIP ORF, than in v545 virus-inoculated animals. All areas of inoculated feet (dorsal, internal, and ventral) appeared less inflamed, with differences in foot thickness measured grossly using calipers correlating with the histopathological findings. At low power, the presence of the GPCMV MIP gene correlated with a much more intense local inflammatory response, and differences in cellularity with an increased mononuclear cell infiltrate were readily discernable. 

### 3.2. Immunogenicity and Safety of v545 Employed as an Attenuated Vaccine Candidate

Based on its favorable attenuation profile in nonpregnant animals, we studied the MIP deletion virus as a candidate live, attenuated vaccine. A two-dose immunization series (5 × 10^4^ pfu) was administered subcutaneously to 15 young, GPCMV-seronegative guinea pigs, at three-week intervals. A similar group of 15 guinea pigs were immunized with phosphate-buffered saline (negative control group). Analysis of ELISA response indicated that only 2/15 animals (13%) demonstrated a response within three weeks of the first vaccination; however, within two weeks of the second vaccination, all animals were GPCMV-seropositive (mean reciprocal ELISA titer, 616; Figure 5(a)). Western blotting was performed in order to further characterize the humoral response following vaccination with v545 virus. These analyses demonstrated (Figure 5(b)) similar patterns of response to those noted using a high-titer polyclonal, anti-GPCMV antisera obtained from an animal infected with wild-type virus.

### 3.3. Maternal and Fetal Outcomes in a Pregnancy/Challenge Study Using v545 Vaccine

The effect of the 545 attenuated virus vaccine on pup mortality due to disseminated GPCMV in Hartley guinea pigs (two doses of vaccine) was analyzed. All 15 dams in the vaccine group completed pregnancy. In the control group, 13 animals became pregnant and had evaluable pregnancy outcomes. In the control group, pup mortality was 35/50 (70%). In contrast, pup mortality in litters born to v545-vaccinated dams was 8/52 (14%; *P* < 0.0001 compared to vaccine group). Mean birth weights of the pups delivered in each group were also compared. Among pups born in the control group, live-born pups (*n* = 15) had a mean weight of 87.1 g (4.25 SE), while dead pups (*n* = 35) had a mean weight of 62.2 g (8.70 SE). In contrast, in the v545 vaccine group, live-born pups (*n* = 44) had an average weight of 97.4 g (2.16 SE), while stillborn pups had a mean weight of 107.6 g (3.97 SE) (*n* = 7; one stillborn pup was not weighed). Overall, mean pup weight was 71.2 g (6.13, SE) in the control group, and 98.7 g (1.86, SE) in the vaccine group (*P* < 0.0001; Table 1). Of 15 litters born to vaccinated dams, 3 (5%) had at least one dead pup, while 12/13 litters born to dams in the control group had at least one dead pup (*P* < 0.001 compared to control group, Fisher's exact test).

The v545 vaccine had a substantial impact on maternal viremia and congenital GPCMV infection. All maternal blood samples taken before SG virus challenge were negative by qPCR. Following SG virus challenge, in control dams, 12/13 demonstrated DNAemia at day 5 postinfection, with a mean viral load of 4.4 ± 0.4 log_10_ genomes/mL. In contrast, only 1 of 15 dams immunized with v545 vaccine demonstrated DNAemia on day 5 after salivary gland virus challenge, with the sole positive sample having a viral concentration of 3.5 log_10_ genomes/mL. PCR of DNA purified from pup organs demonstrated congenital GPCMV infection in 24/49 liver homogenates; 19/46 spleen homogenates; and 28/46 lung homogenates. One dead pup in this group was homogenized en bloc (PCR negative). Overall, 35/50 pups (10/15 among live-born pups, and 25/35 among dead pups) in the control group had congenital infection as evidenced by at least one positive tissue PCR (70% overall congenital infection rate) (Table 2). A total of twenty placentas could be retrieved from this group, and all were positive for GPCMV DNA by PCR. In the v545 vaccine group, PCR of DNA purified from pup organs demonstrated congenital GPCMV infection in 6/52 liver homogenates; 2/52 spleen homogenates; and 5/52 lung homogenates. Overall, congenital GPCMV transmission was identified in 9/52 pups (7/44 live-born pups and 2/8 dead pups) for an overall 11% congenital infection rate (*P* < 0.0001 compared to control). A total of 8/34 retrieved placentas were positive for GPCMV DNA (24%; *P* < 0.0001 versus control group). In PCR-positive placentas from the vaccine group, the mean viral load was 2.2 ± 0.3 log_10_ genomes/mg DNA, compared to 5.7 ± 1.0 log_10_ genomes/mg in placentas from control (unvaccinated) animals (*P* < 0.0001, Mann-Whitney test).

## 4. Discussion

In this study, a live, attenuated CMV vaccine was generated, based on deletion of a functional CCCK gene from the GPCMV genome [13, 14], using a *gpt*-based mutagenesis approach. This recombinant virus was highly attenuated for replication in guinea pigs, both in terms of its capacity to elicit systemic infection as well as in its ability to elicit localized inflammation and histopathology following footpad inoculation. In spite of this attenuation, the virus was capable, when administered as a vaccine to nonpregnant female guinea pigs, of eliciting high-titer ELISA antibody responses. Unfortunately, neutralizing titers could not be performed, due to a limitation in available serum, but the magnitude of the ELISA response was comparable to that observed in natural infection, and the ELISA titer has correlated with the neutralizing response in past studies in this model [32]. Vaccinated animals, following establishment of pregnancy, were protected against DNAemia after virulent salivary-gland virus challenge, and their pups were protected both against GPCMV-associated mortality and GPCMV infection. These observations provide further evidence both for a role of virally encoded immune modulation genes in the pathogenesis of infection *in vivo*, as well as for the highly attenuating effect of genetic manipulations designed to delete such genes in the design of live, attenuated vaccines.

The presence of virally encoded mimics of host CCCK genes has been noted for other rodent CMVs. The most extensively studied of these CKs has been the CCCK encoded by the MCMV MCK-1 gene. Mice infected with recombinant MCMVs with mutations in this gene developed less inflammation at the site of inoculation, demonstrated reduced secondary viremia, and had lower viral titers in the salivary glands [25, 33]. Rat CMV (RCMV) encodes a CCCK with similarity to the MCK-1 gene product, and rats infected with deletion mutants had reduced viral loads in the spleen and salivary glands, as well as reduced swelling and macrophage infiltration at the site of virus inoculation [31]. Previous study of the GPCMV CCCK, GPCMV-MIP [13], demonstrated that a mutant deleted of the CK coding sequences was attenuated for its ability to elicit inflammation and hearing loss following direct intracochlear inoculation in guinea pigs [15, 16]. In additional comparisons of the CK deletion virus, v545, with a virus containing the intact CK gene, vAM403, we observed both reduced DNAemia and weight loss following systemic viral challenge (Figure 3) and reduced footpad swelling and histopathology following localized infection (Figure 4) with the deletion virus compared to the CK-intact virus. This suggests that, as with other rodent CMVs, there is an important role for this CK in the pathogenesis of GPCMV infection *in vivo*. 

The concept of using molecular genetic approaches to engineer recombinant CMVs, toward the goal of creating less pathogenic and/or more immunogenic live, attenuated vaccine candidates, has been previously described for both GPCMV [26] and MCMV [19–21, 34]. The targeting of immune modulation genes in vaccine design is of particular appeal given that there are substantial concerns about the potential long-term risks of live, attenuated HCMV vaccines, which could theoretically include latency, oncogenesis, autoimmune disease, and atherosclerosis [35]. In a previous report, the deletion of 3 GPCMV genes with homology to host MHC-I genes, using a bacterial artificial chromosome recombinatorial approach, resulted in a vaccine virus that was rapidly cleared in animals but was nonetheless highly immunogenic and protective in the congenital infection model. The mechanism of immune evasion mediated by the class I gene family is currently under investigation but may be related to impairment of NK cell clearance. In the present study, *gpt* mutagenesis was chosen to generate recombinant virus, since the location of the GPCMV MIP gene was near the site of the BAC insertion in the viral genome in the BAC construct [23], making additional insertions and modifications in this region more challenging. Presumably, the mechanism of attenuation of the v545 (CCCK deletion) virus described in this study stems from its decreased propensity to mediate an acute inflammatory response. Since the ability of virus to disseminate and establish latency in the salivary gland may be impaired by deletion of viral CKs [25, 31], vaccine strains generated with such targeted deletions may have an improved safety profile.

One limitation of the analyses of the GPCMV MIP knockout and wild-type viruses performed in these studies is that the deletion of the MIP gene was superimposed on a tissue culture-derived (ATCC) viral stock of GPCMV. Subsequent to the initiation of our studies, it was demonstrated that the ATCC stock of GPCMV has a 1.6 kb deletion that removes several GPCMV genes, including *GP129*, *GP131, *and* GP133 *[30, 36]. These genes encode homologs to HCMV UL128, UL130, and UL131, respectively, proteins which play a critical role in formation of the pentameric complex (PC), along with gpUL75 [gH] and gpUL115 [gL], essential for HCMV for the endocytic entry pathway required for infection of endothelial and epithelial cells [37–40]. Recently it has been shown that the GPCMV homologs similarly encode a PC that plays a role in virus entry [41]. Viruses lacking this 1.6 kb region are impaired for replication *in vivo* following experimental challenge of guinea pigs compared to those that retain these gene products [30, 36]. Since our recombinant viruses were generated against an ATCC background lacking this 1.6 kb region [23, 42], the deletion of GPCMV MIP in the recombinant virus, v545, is therefore superimposed upon a virus already lacking in genes that play an important role in the pathogenesis of infection. However, it is clear that the deletion of GPCMV MIP confers additional attenuation, above that already conferred by deletion of the 1.6 kb pathogenicity locus. Importantly, the attenuation of v545 pathogenicity reported in this and other studies [15, 16] was based upon comparisons to vAM403 virus [23], a GPCMV recombinant that is also lacking in this 1.6 kb locus. The vAM403 virus, also engendered by *gpt *mutagenesis, is similar to v545, except that (1) it retains the GPCMV MIP gene; (2) it has the *gpt*/eGFP cassette inserted in a noncoding region of the GPMCV genome. Therefore, the clear pattern of attenuation conferred by deletion of the GPCMV MIP gene in the v545 virus, compared to vAM403, provides reassurance regarding the role of this CCCK in pathogenesis. Efforts are in progress to reengineer the MIP deletion against the backdrop of a full-length GPCMV genome, using bacterial artificial chromosome-based approaches [42]. 

It was of interest to note that the v545 vaccine was capable of eliciting a highly protective immune response, in spite of the fact that it lacks the GP129-133 PC proteins. This observation suggests that the PC is not required for a successful CMV vaccine. Recently, it was shown that, in the absence of the rhesus CMV genes *Rh157.5*, *Rh157.4*, and *Rh157.6* (homologs of HCMV *UL128*,* 130*, and *131*), CD8+ T cells are generated against unusual, diverse, and highly “promiscuous” epitopes [43]. The ability of UL128, 130, and 131 proteins to divert CD8+ T cell targeting away from unconventional epitopes could suggest that deletion of these genes might confer a more diverse T cell response to a CMV vaccine. If similar mechanisms are at play in GPCMV, this could contribute to the efficacy of a live, attenuated vaccine with a deletion in this region of the genome, the lack of an antibody response to the PC notwithstanding. Additional studies of recombinant GPCMVs deleted of key immune evasion and/or pathogenesis genes may shed light on attractive live, attenuated vaccine strategies for consideration for future development in human clinical trials.

## Figures and Tables

**Table 1:** Pregnancy outcomes (pup mortality) after challenge with SG-passaged GPCMV in vaccinated and control dams.

Litter	Dead/total
Control	
1	3/3
2	3/4
3	4/4
4	2/5
5	0/2
6	4/4
7	3/3
8	1/4
9	2/3
10	3/4
11	5/5
12	3/6
13	2/3

Total	35/50 (70%)

V545 vaccine	
1	0/3
2	0/3
3	0/4
4	5/5
5	0/4
6	0/3
7	0/4
8	1/1
9	0/4
10	1/5
11	0/4
12	0/3
13	0/2
14	1/4
15	0/3

Total	8/52 (15%)

**Table 2:** Summary of litters and presence or absence of congenital GPCMV infection in vaccine and control groups.

Litter	PCR positives
Control	
1	3/3
2	4/4
3	4/4
4	4/5
5	2/2
6	2/4
7	3/3
8	1/4
9	2/3
10	4/4
11	1/5
12	4/6
13	1/3

Total	35/50 (70%)

V545 vaccine	
1	0/3
2	0/3
3	1/4
4	2/5
5	0/4
6	0/3
7	0/4
8	0/1
9	1/4
10	0/5
11	0/4
12	0/3
13	1/2
14	1/4
15	3/3

Total	9/52 (17%)
